# Risk of eighteen genome-wide association study-identified genetic variants for colorectal cancer and colorectal adenoma in Han Chinese

**DOI:** 10.18632/oncotarget.12750

**Published:** 2016-10-19

**Authors:** Chunwen Tan, Wangxiong Hu, Yanqin Huang, Jiaojiao Zhou, Shu Zheng

**Affiliations:** ^1^ Cancer Institute (Key Laboratory of Cancer Prevention and Intervention, China National Ministry of Education, Key Laboratory of Molecular Biology in Medical Sciences, Zhejiang Province, China), Second Affiliated Hospital, Zhejiang University School of Medicine, Hangzhou, China; ^2^ Zhejiang Provincial Hospital of Traditional Chinese Medicine, Hangzhou, China

**Keywords:** risk, genetic variants, colorectal cancer, colorectal adenoma, Chinese

## Abstract

**Background:**

Recent genome-wide association studies (GWAS) identified eighteen single-nucleotide polymorphisms (SNPs) to be significantly associated with the risk of colorectal cancer (CRC). However, overall results of the following replications are inconsistent and little is known about whether these associations also exit in colorectal adenomas (CRA).

**Methods:**

The SNP genotyping was performed using a Sequenom MassARRAY to investigate the association of these eighteen SNPs with colorectal neoplasm in a case-control study consisted of 1049 colorectal cancers, 283 adenomas, and 1030 controls.

**Results:**

Two of these SNPs, rs10505477 and rs719725, showed evidence of an association in both CRC and CRA in our study population. Besides, seven SNPs (rs10808555, rs7014346, rs7837328, rs704017, rs11196172, rs4779584, and rs7229639) were significantly associated with CRC, and another one SNP rs11903757 was over-represented in CRA compared with controls. The strongest association was provided by rs11196172 (OR = 2.02, 95% CI = 1.66 - 2.46, *P* < 0.0001) and rs11903757 (OR = 1.96, 95% CI = 1.28 - 3.00, *P* = 0.0026).

**Conclusion:**

These results suggest that some previously reported SNP associations also have impact on CRC and CRA predispositions in the Han Chinese population. A part of genetic risk to CRC is possibly mediated by susceptibility to adenomas.

## INTRODUCTION

Colorectal cancer (CRC) is one of major causes of morbidity and mortality worldwide [1]. In China, CRC is currently the fifth commonest malignancy [2]. Twin studies have clearly shown that inherited susceptibility accounts for about 35% of variance in CRC risk [3]. According to the classical adenoma-carcinoma cascade model, genetic mutation is a critical accelerant contributing to malignant transformation and progression of CRC [4]. However, high-penetrance susceptibility mutations are supposed to explain only < 5% of CRC cases, which suggests the remaining inheritance lies in common low-penetrance variants [5].

The application of genome-wide association studies (GWAS) has made high-throughput genotyping of such common genetic variants possible. Several recent GWAS have uncovered multiple common single nucleotide polymorphisms (SNP) to CRC risk [6–11]. Among these SNPs, rs10808555, rs719725, rs11632715, rs4813802, and rs3824999 are almost exclusively performed in Caucasian and little or nothing is known about the role of such variants in other populations. Moreover, the results from subsequent replication studies of the associations between CRC risk and other seven SNPs rs7014346, rs4939827, rs4779584, rs961253, rs11903757, rs16969681, and rs10505477 are not always concordant [12–14]. Besides, the association between six newly identified CRC risk variants in Asians (rs704017, rs11196172, rs10849432, rs12603526, rs7837328, and rs7229639) and colorectal neoplasm predisposition in Han Chinese is still unclear [8,15]. Investigating their role in CRC risk susceptibility in other population is extremely crucial since the effect of a risk variant might differ greatly between populations [16].

The large majority of CRC develop from adenomas [17]. It has been reported that much of genetic risk to CRC is likely to be mediated in part by predisposition to adenomas [18]. CRC-related SNPs might act through increasing the risk of CRC, CRA, or both.

Thus, to capture a broader spectrum of CRC carcinogenesis, our subjects included both CRC patients and CRA patients. Then we conducted a case-control study to clarify the association between eighteen GWAS identified CRC risk related SNPs and colorectal neoplasm, both colorectal cancer and adenoma, in Han Chinese. We identified seven CRC-predisposing SNPs, one CRA-predisposing SNP and two as both.

## RESULTS

### Subject characteristics

The characteristics of 1049 CRC, 283 CRA, and 1030 controls were detailed in Table 1. CRC cases were older on average and more likely to be male (*P*<0.05). Of the CRC cases, 557 had colon cancer, 482 had rectal cancer, and 10 had cancer in both sites. Regarding histological differentiation, 118, 615, and 78 cases were classified as high, intermediate and low grade, respectively, with the remaining 238 cases were unclear. As for the tumor stage, 147, 362, 359, and 181 cases were classified as stage I, II, III, and IV, respectively. Of the 283 cases with colorectal adenoma, 110 had at least one advanced adenoma.

**Table 1:** The characteristics of the study subjects

	CRC (n=1049) No.(%)	CRA (n=283) No.(%)	Controls (n=1030) No.(%)
Sex			
Male	630(60%)	166(59%)	411(40%)
Female	419(40%)	117(41%)	619(60%)
	*P*<0.0001	*P*<0.0001	
Age	60.9±11.0	58.1±8.6	59.7±8.2
	P=0.006	P=0.006	
Tumor site			
Colon	557(53%)		
Rectum	482(46%)		
Colon and rectum(synchronous)	10(1%)		
Histological grade			
High	118(11%)		
Intermediate	615(59%)		
Low	78(7%)		
Unknow	238(23%)		
Stage			
I	147(14%)		
II	362(35%)		
III	359(34%)		
IV	181(17%)		
Metastases			
Yes	868(%)		
No	181(%)		
Risk of adenomas			
Advanced		110(39%)	
Non-advanced		173(61%)	

### Association between individual SNPs with CRC and CRA risk

The genotype of the eighteen SNPs in the cases and controls were shown in Table 2-3. Remarkably, we found the frequencies of genotypes of rs10505477 were significantly associated with the risk of CRC (CT genotype: OR = 1.50, 95% CI = 1.23-1.84, TT genotype: OR = 1.56, 95% CI = 1.21-2.02) as well as CRA with an OR of 1.44 (95% CI = 1.08-1.92) for CT genotype under dominant model. For another SNP, rs719725, the AC genotype was also associated with an increasing risk of both CRC (OR = 1.27, 95% CI = 1.04-1.54) and CRA (OR = 1.37, 95% CI = 1.03-1.82). Besides, we found the G allele of rs11196172 showed a strong association with the risk of CRC. Subjects with the rs11196172 AG or rs11196172 GG genotype had an OR of 2.02 (95% CI, 1.66-2.46) or 1.83 (95% CI, 1.31-2.56) compared with rs11196172 AA genotype. While the rs10808555 AG (OR = 1.26, 95% CI = 1.05-1.53) but not GG genotype (OR = 1.21, 95% CI = 0.89-1.64) had an increase risk compared with the rs10808555 AA genotype. An obvious increased risk was observed among individuals with rs7014346 GA genotype (OR = 1.29, 95% CI = 1.07-1.55), however, the rs7014346 AA was not associated with the risk (OR = 1.21, 95% CI = 0.89-1.64). Likewise, rs7837328 GA and rs704017 AG genotype had an OR of 1.48 (95% CI = 1.20-1.82) and 1.21 (95% CI = 1.01-1.46), respectively. The G allele of rs7229639 increase the likelihood of CRC only in a dominant genetic model (OR = 1.23, 95% CI = 1.02-1.48). For rs4779584, the TC genotype was associated with a significantly decrease in the risk of CRC, with an OR of 0.70 (95% CI = 0.57-0.86) and this is the only protective factor in our study.

**Table 2:** Association of 18 SNPs with CRC risk in Chinese

location^a^	rsID	CAU^b^	CHB^b^		GWAS^f^			none risk /risk allele	MAF controls	MAF CRC	Colorectal cancer			
		miror allele	miror allele	MAF	OR	*P*-value	Ref ^g^				OR(95%CI)			*P*-value^d^
											Aa	aa	dominant model	
2q32.2	rs11903757^e^	C	C	0.211	1.15	4.06E-06	1	T/C	0.04	0.04	1.01 (0.72-1.42)	NA	NA	NA
8q24.21	rs10505477	T	T	0.460	1.16	5.05E-04	2	C/T	0.40	0.46	**1.50 (1.23-1.84)**	**1.56 (1.21-2.02)**	**1.52 (1.25-1.84)**	<0.0001
8q24.21	rs10808555	G	G	0.296	1.12	2.60E-03	3	A/G	0.30	0.33	**1.26 (1.05-1.53)**	1.21 (0.89-1.64)	**1.25 (1.05-1.50)**	0.014
8q24.21	rs7014346	A	A	0.310	1.11	1.60E-02	4	G/A	0.30	0.33	**1.29 (1.07-1.55)**	1.21 (0.89-1.64)	**1.27 (1.06-1.52)**	0.0079
8q24.21	rs7837328	A	A	0.088	1.23	1.42E-03	5	G/A	0.38	0.44	**1.48 (1.20-1.82)**	**1.63 (1.23-2.15)**	**1.52 (1.25-1.85)**	<0.0001
9p24.1	rs719725	C	C	0.408	1.10	2.50E-03	6	A/C	0.30	0.34	**1.27 (1.04-1.54)**	1.21 (0.89-1.64)	**1.26 (1.04-1.51)**	0.015
10q22.3	rs704017	G	A	0.477	1.10	9.99E-04	7	A/G	0.27	0.29	**1.21 (1.01-1.46)**	0.95 (0.67-1.36)	1.17 (0.98-1.40)	0.079
10q25.2	rs11196172	NA^c^	NA	NA	1.14	5.18E-07	7	A/G	0.31	0.41	**2.02 (1.66-2.46)**	**1.83 (1.31-2.56)**	**1.99 (1.65-2.41)**	<0.0001
11q13.4	rs3824999	C	C	0.487	1.20	2.42E-02	8	A/C	0.39	0.40	1.10 (0.91-1.34)	1.08 (0.83-1.40)	1.10 (0.91-1.32)	0.33
12p13.31	rs10849432	C	NA	NA	1.14	6.95E-06	7	C/T	0.20	0.21	0.96 (0.79-1.16)	1.30 (0.84-2.03)	0.99 (0.83-1.19)	0.94
15q13.3	rs16969681	NA	T	0.364	0.93	1.81E-02	9	C/T	0.41	0.41	1.01 (0.83-1.24)	1.02 (0.78-1.32)	1.02 (0.84-1.22)	0.87
15q13.3	rs11632715	NA	G	0.233	1.05	1.17E-02	9	A/G	0.17	0.17	0.94 (0.77-1.14)	0.99 (0.61-1.61)	0.94 (0.78-1.14)	0.54
15q13.3	rs4779584	C	T	0.168	1.23	4.70E-07	10	C/T	0.20	0.15	**0.70 (0.57-0.86)**	0.64 (0.39-1.05)	**0.69 (0.57-0.84)**	0.0002
17p13.3	rs12603526	C	C	0.013	1.10	3.80E-04	7	T/C	0.26	0.27	1.11 (0.92-1.34)	0.93 (0.64-1.35)	1.09 (0.91-1.29)	0.37
18q21.1	rs4939827	T	T	0.473	0.90	8.87E-04	11	C/T	0.28	0.29	0.98 (0.82-1.18)	1.14 (0.81-1.63)	1.01 (0.84-1.20)	0.94
18q21.1	rs7229639	A	A	0.088	1.20	6.65E-05	11	A/G	0.17	0.20	1.21 (1.00-1.47)	1.44 (0.88-2.37)	**1.23 (1.02-1.48)**	0.028
20p12.3	rs4813802	G	G	0.376	1.05	2.50E-02	1	T/C	0.23	0.25	1.18 (0.98-1.43)	1.15 (0.79-1.68)	1.18 (0.98-1.41)	0.074
20p12.3	rs961253	A	A	0.403	1.11	4.60E-05	12	C/A	0.08	0.08	1.03 (0.81-1.32)	0.59 (0.17-2.04)	1.01 (0.79-1.29)	0.93

a SNP locations based on Human Genome build 36.

b Minor allele and minor allele frequencies of Caucasian and Han Chinese in Beijing from HapMap Release 28

c not available

d Adjusted for age sex

e OR and 95%CI for rs11903757 TC VS TT genotype in our study

f results in validation stage

g References:1.Ulrike Peters et al. Gastroenterology 2012; 2.Brent W Zanke et al. Nature Genetics 2007;3. Richard S Houlston et al. Nature Genetics 2008; 4.Wei-Hua Jia et al.Nature Genetics 2013;5.R Cui et al.Gut 2011;6.Brent W et al.Zanke et al. Nature Genetics 2007;7.Ben Zhang et al. Nature Genetics 2014;8.Luis M. Real et al.PLOS ONE 2014;9.Tomlinson et al. Nature Genetics 2011;10.Ian PM Tomlinson et al. Nature Genetics 2007;11.Ben Zhang et al.International Journal of Cancer 2014; 12. COGENT Nature Genetics 2008.

**Table 3:** Association of 18 SNPs with CRA risk in Chinese

location^a^	rsID	CAU^b^	CHB^b^		none risk/risk allele	MAF controls	MAF CRA	Colorectal adenoma			
		miror allele	miror allele	MAF				OR(95%CI)			*P*-value^d^
								Aa	aa	dominant model	
2q32.2	rs11903757^e^	C	C	0.211	T/C	0.04	0.07	**1.96 (1.28-3.00)**	NA	NA	0.0026
8q24.21	rs10505477	T	T	0.460	C/T	0.40	0.44	**1.50 (1.10-2.03)**	1.28 (0.86-1.92)	**1.44 (1.08-1.92)**	0.013
8q24.21	rs10808555	G	G	0.296	A/G	0.30	0.31	1.17 (0.88-1.55)	1.02 (0.64-1.64)	1.14 (0.87-1.49)	0.34
8q24.21	rs7014346	A	A	0.310	G/A	0.30	0.30	1.15 (0.87-1.52)	0.93 (0.57-1.51)	1.10 (0.84-1.44)	0.47
8q24.21	rs7837328	A	A	0.088	G/A	0.38	0.39	1.25 (0.93-1.68)	0.98 (0.63-1.52)	1.18 (0.89-1.57)	0.24
9p24.1	rs719725	C	C	0.408	A/C	0.30	0.32	**1.37 (1.03-1.82)**	0.91 (0.56-1.49)	1.27 (0.97-1.67)	0.086
10q22.3	rs704017	G	A	0.477	A/G	0.27	0.25	0.92 (0.70-1.22)	0.67 (0.37-1.21)	0.88 (0.67-1.16)	0.36
10q25.2	rs11196172	NA^c^	NA	NA	A/G	0.31	0.36	1.23 (0.92-1.66)	1.58 (0.97-2.57)	1.29 (0.97-1.71)	0.084
11q13.4	rs3824999	C	C	0.487	A/C	0.39	0.41	1.18 (0.88-1.58)	1.11 (0.74-1.65)	1.16 (0.88-1.53)	0.3
12p13.31	rs10849432	C	NA	NA	C/T	0.20	0.19	0.83 (0.61-1.11)	1.13 (0.57-2.22)	0.86 (0.65-1.13)	0.28
15q13.3	rs16969681	NA	T	0.364	C/T	0.41	0.39	1.16 (0.86-1.56)	0.72 (0.46-1.12)	1.04 (0.78-1.39)	0.77
15q13.3	rs11632715	NA	G	0.233	A/G	0.17	0.18	1.15 (0.86-1.55)	0.80 (0.36-1.77)	1.11 (0.84-1.48)	0.46
15q13.3	rs4779584	C	T	0.168	C/T	0.20	0.18	0.85 (0.63-1.14)	0.78 (0.38-1.61)	0.84 (0.63-1.12)	0.24
17p13.3	rs12603526	C	C	0.013	T/C	0.26	0.27	1.20 (0.91-1.59)	0.84 (0.46-1.53)	1.15 (0.88-1.50)	0.31
18q21.1	rs4939827	T	T	0.473	C/T	0.28	0.30	0.97 (0.73-1.29)	1.57 (0.96-2.55)	1.05 (0.81-1.38)	0.71
18q21.1	rs7229639	A	A	0.088	A/G	0.17	0.20	1.21 (0.91-1.61)	1.17 (0.53-2.54)	1.21 (0.91-1.60)	0.19
20p12.3	rs4813802	G	G	0.376	T/C	0.23	0.25	1.03 (0.77-1.37)	1.31 (0.78-2.21)	1.07 (0.82-1.41)	0.62
20p12.3	rs961253	A	A	0.403	C/A	0.08	0.09	1.06 (0.74-1.53)	1.38 (0.36-5.33)	1.08 (0.75-1.54)	0.69

a SNP locations based on Human Genome build 36.

b Minor allele and minor allele frequencies of Caucasian and Han Chinese in Beijing from HapMap Release 28

c not available

d Adjusted for age sex

e OR and 95%CI for rs11903757 TC VS TT genotype in our study

Among the CRA risk associated genetic variants, the TC genotype of rs11903757 was associated with an increasing risk of CRA (OR = 1.96, 95% CI = 1.28-3.00), whereas no heterozygous variants carrier was found in our population. There were no significant associations between other SNPs and risk of CRC or CRA.

The four SNPs genotyped in the 8q24.21 region within 17 kb showed high linkage disequilibrium (*r*^2^ range: 0.97-0.99). Haplotype analysis revealed that the haplotype containing the variant allele at rs10505477, rs10808555, rs7837328, and rs10505476 were associated with an increase risk of CRC (OR = 1.251-1.487).

### Association between SNPs and clinical characteristics

Further more, we assessed the associations between SNP genotype and clinical characteristics (Table 4-6). After stratification, four SNPs (rs10505477, rs7837328, rs11196172, and rs4779584) were associated with the cancer risk in rectum compared with keeping the association with cancer risk in colon under dominant model. Furthermore, we found a significant histological grade-specific difference in risk for six SNPs: rs10505477, rs10808555, rs7014346, rs7837328, rs4779584, and rs4813802. All of them were significantly associated with increased risk of low or intermediate histological grade, while rs719725, and rs11196172 also presented significantly elevated risk in high histological grade. In the case of tumor stage, rs10505477, rs719725, rs11196172, and rs4779584 were significantly associated with stage I or II disease, whereas eight SNPs (rs10505477, rs10808555, rs7014346, rs7837328, rs704017, rs11196172, rs4779584, and rs7229639) were significantly associated with stage III or IV. High-risk allele of rs10505477, rs7014346, rs7837328, rs11196172, and rs4813802 were more frequently found in patients with metastatic CRC. However, all of the CRC risk variants mentioned before showed significant associations with non-metastatic CRC. Among all these CRC risk SNPs, we found rs10808555, rs7014346, and rs7837328 seem to significantly increase risk for being more aggressive CRC.

**Table 4:** Association of 18 SNPs with tumor site and histological grade of CRC

rsID	Tumor site				Histological grade			
	colon		Rectum		High		Low/Intermediate	
	OR(95%CI)	*P*-value	OR(95%CI)	*P*-value	OR(95%CI)	*P*-value	OR(95%CI)	*P*-value
rs11903757^a^	0.99 (0.65-1.49)	0.95	1.02 (0.67-1.56)	0.93	1.33 (0.69-2.60)	0.41	0.90 (0.61-1.33)	0.59
rs10505477	**1.47 (1.17-1.84)**	0.001	**1.59 (1.25-2.04)**	0.0001	1.27 (0.84-1.92)	0.25	**1.58 (1.27-1.96)**	<0.0001
rs10808555	**1.31 (1.05-1.62)**	0.014	1.23 (0.98-1.54)	0.074	1.23 (0.83-1.82)	0.31	**1.26 (1.03-1.54)**	0.024
rs7014346	**1.33 (1.07-1.64)**	0.0092	1.24 (0.99-1.55)	0.06	1.25 (0.85-1.84)	0.26	**1.26 (1.03-1.54)**	0.022
rs7837328	**1.49 (1.17-1.89)**	0.0009	**1.54 (1.20-1.97)**	0.0007	1.27 (0.83-1.95)	0.27	**1.58 (1.26-1.98)**	0.0001
rs719725	**1.41 (1.13-1.76)**	0.0024	1.15 (0.91-1.44)	0.25	**1.58 (1.05-2.39)**	0.027	**1.26 (1.03-1.56)**	0.028
rs704017	1.10 (0.89-1.36)	0.39	**1.26 (1.01-1.58)**	0.04	0.92 (0.62-1.36)	0.68	1.19 (0.98-1.45)	0.084
rs11196172	**2.12 (1.68-2.68)**	0.0001	**1.92 (1.51-2.45)**	<0.0001	**1.71 (1.12-2.62)**	0.011	**2.11 (1.70-2.62)**	<0.0001
rs3824999	1.10 (0.89-1.37)	0.38	1.09 (0.87-1.38)	0.45	1.02 (0.68-1.52)	0.92	1.13 (0.92-1.39)	0.25
rs10849432	1.02 (0.82-1.27)	0.87	0.97 (0.77-1.22)	0.8	1.03 (0.69-1.53)	0.88	0.99 (0.81-1.22)	0.96
rs16969681	0.89 (0.71-1.11)	0.28	1.19 (0.94-1.52)	0.14	0.74 (0.49-1.10)	0.14	1.06 (0.86-1.31)	0.57
rs11632715	0.87 (0.69-1.10)	0.24	1.02 (0.81-1.30)	0.86	1.01 (0.67-1.52)	0.97	0.85 (0.69-1.06)	0.15
rs4779584	**0.71 (0.56-0.89)**	0.0033	**0.69 (0.54-0.88)**	0.0026	0.73 (0.48-1.11)	0.13	**0.71 (0.57-0.88)**	0.0017
rs12603526	1.16 (0.94-1.44)	0.16	1.01 (0.81-1.27)	0.9	1.40 (0.96-2.06)	0.083	1.07 (0.87-1.30)	0.53
rs4939827	0.96 (0.78-1.18)	0.68	1.08 (0.86-1.35)	0.5	1.15 (0.78-1.68)	0.49	1.06 (0.87-1.29)	0.57
rs7229639	**1.38 (1.11-1.72)**	0.004	1.06 (0.84-1.35)	0.61	0.99 (0.65-1.50)	0.96	1.17 (0.95-1.45)	0.13
rs4813802	1.12 (0.90-1.39)	0.3	**1.31 (1.05-1.64)**	0.019	1.00 (0.68-1.49)	0.99	**1.35 (1.11-1.66)**	0.003
rs961253	0.97 (0.73-1.30)	0.85	1.01 (0.75-1.38)	0.93	0.91 (0.53-1.57)	0.73	0.95 (0.72-1.25)	0.72

All the OR and 95%CI were computed under a dominant genetic model except for rs11903757.

a OR and 95%CI for rs11903757 TC VS TT genotype

**Table 5:** Association of 18 SNPs with tumor stage and metastatic status of CRC

rsID	Tumor stage				Metastases disease			
	I and II		III and IV		stage IV		not stage IV	
	OR(95%CI)	*P*-value	OR(95%CI)	*P*-value	OR(95%CI)	*P*-value	OR(95%CI)	*P*-value
rs11903757^a^	1.10 (0.74-1.66)	0.63	0.93 (0.61-1.42)	0.73	1.03 (0.56-1.91)	0.92	1.00 (0.70-1.42)	0.99
rs10505477	**1.33 (1.05-1.68)**	0.017	**1.75 (1.38-2.22)**	<0.0001	**2.00 (1.38-2.91)**	0.0001	**1.44 (1.18-1.76)**	0.0003
rs10808555	1.12 (0.90-1.40)	0.3	**1.42 (1.14-1.76)**	0.0018	1.38 (0.99-1.92)	0.057	**1.23 (1.02-1.49)**	0.029
rs7014346	1.16 (0.93-1.44)	0.19	**1.40 (1.13-1.74)**	0.0019	**1.51 (1.09-2.10)**	0.012	**1.23 (1.02-1.48)**	0.03
rs7837328	1.25 (0.98-1.58)	0.071	**1.88 (1.47-2.42)**	<0.0001	**2.05 (1.39-3.03)**	0.0002	**1.43 (1.16-1.76)**	0.0007
rs719725	**1.40 (1.11-1.76)**	0.0042	1.16 (0.92-1.45)	0.2	1.35 (0.96-1.91)	0.083	**1.23 (1.01-1.49)**	0.036
rs704017	1.08 (0.87-1.35)	0.47	**1.26 (1.01-1.56)**	0.036	1.29 (0.94-1.78)	0.11	1.14 (0.95-1.37)	0.17
rs11196172	**2.08 (1.64-2.66)**	<0.0001	**1.93 (1.53-2.44)**	<0.0001	**2.65 (1.81-3.87)**	<0.0001	**1.89 (1.55-2.31)**	<0.0001
rs3824999	1.00 (0.80-1.25)	0.99	1.20 (0.96-1.50)	0.11	1.28 (0.91-1.79)	0.16	1.06 (0.88-1.29)	0.53
rs10849432	1.05 (0.84-1.32)	0.67	0.94 (0.75-1.17)	0.58	0.92 (0.66-1.29)	0.63	0.99 (0.82-1.20)	0.93
rs16969681	0.98 (0.78-1.24)	0.88	1.04 (0.83-1.30)	0.75	1.01 (0.72-1.42)	0.94	1.01 (0.83-1.23)	0.9
rs11632715	1.09 (0.86-1.38)	0.46	0.80 (0.63-1.01)	0.062	**0.63 (0.43-0.91)**	0.012	1.02 (0.83-1.24)	0.86
rs4779584	**0.70 (0.55-0.89)**	0.0033	**0.70 (0.55-0.88)**	0.0026	0.76 (0.53-1.07)	0.11	**0.69 (0.56-0.85)**	0.0003
rs12603526	1.17 (0.94-1.45)	0.17	1.01 (0.82-1.25)	0.92	0.74 (0.53-1.02)	0.065	1.17 (0.98-1.41)	0.09
rs4939827	0.98 (0.79-1.23)	0.89	1.03 (0.83-1.27)	0.8	0.94 (0.69-1.30)	0.72	1.02 (0.85-1.23)	0.81
rs7229639	1.16 (0.92-1.46)	0.21	**1.31 (1.05-1.63)**	0.019	1.28 (0.92-1.78)	0.15	**1.22 (1.01-1.49)**	0.043
rs4813802	1.20 (0.96-1.49)	0000.11	1.19 (0.96-1.48)	0.11	**1.42 (1.03-1.96)**	0.032	1.15 (0.95-1.38)	0.16
rs961253	1.00 (0.74-1.35)	0.99	1.01 (0.76-1.36)	0.93	1.05 (0.67-1.62)	0.84	1.00 (0.78-1.30)	0.97

All the OR and 95%CI were computed under a dominant genetic model except for rs11903757.

a OR and 95%CI for rs11903757 TC VS TT genotype

**Table 6:** Association of 18 SNPs with advanced and non-advanced CRA

rsID	Risk of adenomas			
	Advanced		Non-advanced	
	OR(95%CI)	*P*-value	OR(95%CI)	*P*-value
rs11903757^a^	1.60 (0.84-3.07)	0.17	**2.27 (1.39-3.71)**	0.0018
rs10505477	1.44 (0.93-2.21)	0.096	1.42 (1.00-2.03)	0.048
rs10808555	0.96 (0.64-1.42)	0.82	1.28 (0.92-1.78)	0.14
rs7014346	0.88 (0.59-1.31)	0.52	1.28 (0.92-1.78)	0.14
rs7837328	1.05 (0.69-1.58)	0.83	1.27 (0.90-1.80)	0.18
rs719725	1.15 (0.77-1.73)	0.48	1.36 (0.97-1.90)	0.07
rs704017	0.91 (0.61-1.36)	0.66	0.85 (0.61-1.18)	0.34
rs11196172	**1.60 (1.03-2.50)**	0.034	1.11 (0.78-1.56)	0.56
rs3824999	**1.81 (1.16-2.85)**	0.0072	0.90 (0.65-1.26)	0.55
rs10849432	0.78 (0.51-1.20)	0.25	0.90 (0.64-1.26)	0.53
rs16969681	0.97 (0.64-1.47)	0.88	1.11 (0.78-1.58)	0.57
rs11632715	1.33 (0.88-2.00)	0.19	1.00 (0.70-1.42)	1
rs4779584	0.70 (0.45-1.09)	0.11	0.94 (0.67-1.33)	0.74
rs12603526	1.35 (0.91-2.00)	0.14	1.03 (0.74-1.43)	0.86
rs4939827	1.10 (0.74-1.63)	0.65	1.02 (0.74-1.41)	0.9
rs7229639	1.27 (0.84-1.91)	0.27	1.17 (0.83-1.65)	0.37
rs4813802	1.28 (0.86-1.90)	0.23	0.96 (0.68-1.34)	0.79
rs961253	0.93 (0.54-1.62)	0.8	1.17 (0.77-1.79)	0.47

All the OR and 95%CI were computed under a dominant genetic model except for rs11903757.

a OR and 95%CI for rs11903757 TC VS TT genotype

With regarding to CRA, rs1193757 was only associated with non-advanced CRA and the association between rs11196172 and rs3824999 and colorectal adenoma risk was limited to advanced stage. Moreover, the effect of risk variants rs11903757 to non-advanced adenoma is more pronounced than that to overall adenoma, with an OR of 2.27 (95% CI = 1.39-3.71).

## DISCUSSION

Despite that a number of genetic variants associated with CRC risk have now been identified, they have been performed almost exclusively in Caucasian populations and the effects of these risk variants in Han Chinese population are as yet unknown [19]. Because the prevalence of CRC, allele frequency of SNPs and linkage disequilibrium (LD) structure differ greatly among populations, it is thus extremely important to known whether these associations still exist in different ethnic populations [20–22]. We, therefore, systematically evaluated the association between eighteen SNPs and colorectal neoplasm risk in CRC as well as CRA to examine the full spectrum of colorectal carcinogenesis. Furthermore, we explored the potential effect of clinic-pathological variables of CRC and CRA on these variant-associated susceptibilities. SNP rs10505477 and rs719725 were found to be significantly associated with both CRC and CRA. In addition, we confirmed SNPs at 8q24.21 10q22.3, 10q25.2, 15q13.3, and 18q21.1 were genetic risk factors for CRC. We also observed the association between SNP from risk loci at 2q32.2 and the risk of CRA (Supplementary Table S1).

### Associations of both colorectal cancer and adenoma risk with individual SNP

SNP rs10505477 on chromosome 8q24.21 was firstly identified as a risk locus for CRC by a GWAS in populations derived from the South America and Scotland [23], and then supported by another two GWAS in the Spanish and the East Asians populations as well as several other population-based studies [6, 24–31]. However, there were various studies failed to confirm the association between rs10505477 and CRC risk [12, 24, 32]. It has also been reported that the locus influences the susceptibility in both CRA and CRC [33]. Although a GWAS identified rs10505477 is the most likely SNP associated with CRA, it did not achieve a genome-wide significant *P*-value [34].

We also found significant association of rs10505477 with both colon and rectum in stratified analysis. In a previous study, the association between this SNP and CRC risk had no difference at tumor site [26]. And similar result held in our study. While Monir *et al*. observed tumor site-specific association of rs10505477 with CRC risk merely in colon [31].

Rs10505477 is near the gene putative POU domain, class 5, transcription factor 1B (*POU5FIB*). Overexpression of this gene has recently been reported in gastric cancer and it can promote tumor formation and growth *in vivo* [35]. But the association was not observed between *POU5FIB* expression and genotype of rs10505477 [36]. This SNP also maps to the intron of a long noncoding RNA (lncRNA), cancer-associated region long noncoding RNAs-1 (*CARLos-1*). Silico studies have shown that rs10505477 change the CARLos-1 local folding structure, which suggests it may lead to aberrant expression of *CARLos-1* [37]. Although the biological function of *CARLos-1* is still unclear, the *CARLos-5*, which is in the same region with *CARLos-1*, has a function in tumor development [38], indicating that *CARLos-1* may also play a role in carcinogenesis.

Another CRC and CRA risk-associated genetic variant rs719725 resides in an intergenic region on chromosome 9p24.1. The most proximal gene, tumor protein D52-like 3(*TPD52L3*), one of three members of human TPD52 (hTPD52), is 37 kb distal to it. hTPD52 is first identified in breast cancer. Moreover, they have been shown to participate in cell proliferation, apoptosis, and vesicle trafficking [39]. This SNP was previously identified as a genetic susceptibility factor for CRC in a GWAS study of various Caucasian populations [40]. Although a majority of subsequent validation attempts were failed, there were still several lines of evidence support its association with CRC risk. However, its role in CRA predisposition has drawn little attention [41–46]. Merely marginally significant evidence for the association between rs719725 and CRA risk was observed in a case-control study [47]. In our study, respectively compared with rs10505477 CC or rs719725 AA genotype, individuals who carried rs10505477 CT / TT or rs719725 AC genotype had elevated risk in colorectal carcinoma as well as adenoma, suggesting the possibility that the association between these two SNPs and CRC susceptibility is mediated through adenoma risk, namely the SNP might involve in initiation of tumor only, or might still have an effect at the progression stage, or both.

### Colorectal cancer susceptibility genetic variants

Three SNPs rs10808555, rs7837328, and rs7014346, all mapping to 8q24.21, were associated with CRC susceptibility. This finding is consistent with a meta-analysis of genome-wide association data [7]. Moreover, after stratified by tumor differentiation, stage, presence or absence of distant metastasis, we found an association of these three SNPs with aggressive advanced cancer, providing evidence that they play a role in tumor initiation and development. The identification of rs10808555 as a risk variant for CRC was confirmed by a study in Caucasians [33]. And SNP rs7837328 had been identified as a susceptibility variant of prostate cancer in various studies [48–50]. The finding that colorectal epithelial cell proliferation was higher with the presence of either rs10808555 GG genotype or rs7837328 AA genotype suggested a plausible linkage of the CRC risk association [13]. Just as rs10505477, SNP rs7014346 also resides near *POU5FIB*. A study confirmed the association between rs7014346 and the susceptibility of CRC in Hong Kong Chinese, although this association was not observed in other two studies in Chinese population [51, 52].

We further determined the haplotype block structure across 8q24.21 containing the variant allele at rs10505477, rs10808555, rs7837328, and rs10505476 to capture information on the LD structure in the region, and hence potentially provide greater power to detect association. Haplotype analysis of these variants showed that the haplotype containing rs10505477, rs10808555, rs7837328, and rs10505476 were associated with an increased risk of CRC (Table 7). Howerer, the strength of the association for the haplotype (OR_per T-A-G-G haplotype_ = 1.487) was not greater than that of single variant rs10505477 and rs7837328 (OR = 1.52 for both under dominant model) while slightly larger than previous haplotype analysis in this region (OR=1.17) [33].

**Table 7:** Association of CRC risk with the haplotypes comprising rs10505477, rs10808555, rs7837328, and rs7014346

Haplotype^a^	Frequency		*P*^b^	OR (95% CI)
	Case	Controls		
CAGG	0.527	0.594		1.000 (Reference)
TGAA	0.327	0.292	0.002	1.251 (1.088-1.438)
TAAG	0.084	0.068	0.008	1.385 (1.088-1.763)
TAGG	0.048	0.035	0.015	1.487 (1.081-2.047)

a Haplotypes of rs10505477, rs10808555, rs7837328, and rs7014346

b *P* values from unconditional logistic regression analyses, adjusted for age and gender.

Another two SNPs located on 10q22.3 (rs704017) and 10q25.2 (rs11196172) were new loci associated with CRC observed by a recent large scale genetic study in East Asians, yielded an OR of 1.10 (95%CI = 1.10-1.18) and 1.14 (95%CI = 1.10-1.18) respectively based on the risk allele [8]. SNP rs704017 maps within the third intron of the gene *ZMIZ1* antisense RNA 1 (ZMIZ1-AS1), which is a miscRNA and its function is still unknown. In our study, rs11196172 GG genotype associated with 2.02-fold significantly increase of CRC risk (95% CI = 1.66-2.46, *P* < 0.0001). Besides, stratified analysis provided evidence that this genetic variant also associated with advanced adenoma risk. It suggested that this SNP may participate in late stage of adenoma and carcinoma genesis. The rs11196172 SNP is located in the *TCF7L2* gene. TCF7L2 is a key transcription factor in Wnt/β-catenin signal transduction pathway. It had been reported that in colon tumor tissue the G allele of rs11196172 was significantly related with increased expression of *TCF7L2* [8]. Inhibition of TCF7L2 in CRC cells results in cell cycle arrest and differentiation, induces cell apoptosis while inhibits cell proliferation [53, 54].

Rs4779584 is proximal to the *GREM1* locus on chromosome 15q13.3, and the association between this SNP and CRC susceptibility had been validated in Hong Kong and Taiwan Chinese [55, 56]. It was reported that rs4779584 is also associated with CRA risk [57–59]. But this association was not observed in the Han Chinese population we studied. *GREM1* encodes a member of the bone morphogenic protein (BMP) antagonist family. It is an essential component in the transforming growth factor-β (TGF-β) pathway. Moreover, the TGF-β/BMP pathway has been demonstrated implicated in colorectal carcinogenesis. GREM1 promote the loss of cancer cell differentiation [60]. A recent research found that aberrant expression of epithelial GREM1 initiates colonic tumorgenesis [61]. This indicates that GREM1 may be a possible explanation for the association between rs4779584 and CRC risk.

Another SNP in the TGF-β family signaling pathway, rs7229639, was also found to be associated with CRC risk. This SNP is located in the third intron of the *SMAD 7* gene, which is a key member in TGF-β pathway. The association of this variant with CRC risk was initially observed through a GWAS conducted in East Asians in 2014 [62]. In the stratified analysis of this study, an OR of 1.20 (*P* = 6.65×10^−5^) per-allele was yielded in Chinese and for the first time the association between this SNP and CRC was replicated. Our similar finding further validated the association between this variant and the susceptibility of CRC in Chinese.

Little evidence showed genetic involvement of these seven CRC predisposition variants in CRA risk suggests that they might only affect the malignant stage of colorectal carcinogenesis in our study population.

### Colorectal adenoma risk related variants

Interestingly, we found SNP rs11903757 was associated with CRA but not CRC risk. Previous studies of rs11903757 focused on CRC risk, but the overall results were inconclusive and little is known about its role in CRA susceptibility. In our study, TC genotype of rs11903757 showed the most significantly association of CRA risk with an OR of 1.96 (95% CI = 1.28-3.00, *P*=0.0026). This SNP lies closely to the region of the gene nucleic acid binding protein 1 (*NABP1*), which encodes a single-stranded DNA (ssDNA)-binding protein and plays a critical role in genomic stability and participates in DNA damage response [63, 64]. Functional experiments are needed to identify the causal loci.

In this study, neither CRC nor CRA predisposition are related with previously identified SNPs: rs2287939, rs10849432, rs7229639, and rs961253. The failure to replicate Caucasian (CAU)-identified variants could be due to clear difference in terms of allele frequency present between CAU and Han Chinese in Beijing (CHB) (Table 2) or different pattern of linkage dis-equilibrium. Another reason for the disparity might lie in the gene-environment interaction. It is also possible that the association is real but the gene effect size is too weak to be detected with sufficient statistical power.

Recently, a GWAS of colorectal cancer in East Asians [65] and two in Chinese (labeled as Chinese 1 [66] and Chinese 2 [67]) identified new novel loci for colorectal cancer risk. Most of our variants were included in the three GWAS (Supplementary Table S2). However, the majority of them failed to reach statistical significance. We noticed that study subjects of the GWAS in East Asians and in Chinese 2 were enrolled mainly from the three largest cosmopolitan cities of China: Beijing, Shanghai, and Guangzhou. It was also notable that the minor allele of rs4779584 was C in our population but T in these studies. Genetic heterogeneity on account of sampling from a mixture of migrant workers from all over China would probably cause different study findings. In addition, for GWAS in Chinese 2, almost every interesting associations of our study demonstrated *P* values of less than 0.05. These variants did not reach statistical significance of GWAS possibly due to insufficient sample size, with only 1,049 colorectal cancer cases and 1,315 controls in the GWAS stage. These could be the explanation for the disparity.

Our study has several limitations. First, our sample size, especially for CRA, was relative small and the subjects were not representative of the general Chinese population. Second, carcinogenic exposure which may modify the effects of the genetic factors was not taken into consideration. Third, the bioinformatics analysis we performed is insufficient for clarifying a direct causal association. Therefore, the interpretation of our results should be taken with caution.

In summary, our case-control study provide convincing evidence for assigning seven SNPs as CRC-predisposing, one as CRA-predisposing and two as both. These variations may have potential implications for modulating colorectal neoplasm screen measures and improve our understanding of the initiation and progression of CRC. Further studies are warranted to characterize functional sequences that cause carcinogenesis and identify novel variants contributing to susceptibility of colorectal tumor.

## MATERIALS AND METHODS

### Study subjects

All participating individuals were genetically unrelated Han Chinese. The characteristics of CRC, CRA, and controls included in this study were summarized in Table 1. CRC patients were recruited between 2011 and 2013 in the Second Affiliated Hospital, School of Medicine, Zhejiang University, China. CRA and controls were enrollees of a colorectal cancer screening programme conducted in the same regions and underwent a colonoscopy during the same time period. The diagnosis of CRC and CRA were confirmed histologically without any treatment before. Individuals with negative colonoscopic findings and no previously recorded or currently diagnosed CRC or CRA were defined as the controls of this study. The exclusion criteria of all subjects included a family history of CRC, histories of previous cancers, genetic colorectal cancer syndromes (i.e. familial adenomatous polyposis), bowel resection, inflammatory bowel disease, and partial colonoscopy. Based on colonoscopy and pathology reports, adenomas were defined as advanced if they 1) were ≥1cm in diameter or 2) had ≥20% villous components or showing high-grade dysplasia. Written informed consent was obtained from each subject before collection of blood samples and information. This study was approved by the Institutional Review Board of Clinical Research, the Second Affiliated Hospital, Zhejiang University School of Medicine and complete in accordance with the ethical principles in declaration of Helsinki (Oct 2008).

### Genotyping

Blood samples collected from each study subject were stored at -80°C before DNA extraction. Genomic DNA was isolated from peripheral blood leukocytes, using the DNA Isolation Kit for Mammalian Blood (Roche, Indianapolis, USA). Genotyping of the selected SNPs was performed using the Sequenom MassARRAY platform with iPLEX Gold chemistry on a matrix-assisted laser desorption/ ionization time-of-flight mass spectrometer (Sequenom, San Diego, California) according to the supplier's instructions. The extension primers were designed using MassARRAY Assay Design 4.0 software (Sequenom, San Diego, California). Products generated from the polymerase chain reaction (PCR) were transferred to 384-well Spectro-CHIPs (Sequenom, San Diego, California), and analyzed in a Compact Mass Spectrometer, using the MassARRAY Typer 4.0 Software. The PCR assay was arrayed with positive and negative controls and duplicated samples in each 384-well format as quality control. All the genotyping results were generated and checked by laboratory staff blinded to the sample status.

### Statistical analysis

Differences in demographic variables were examined by the *X*^2^ test and *t*-test. All the eighteen SNPs were tested for the Hardy-Weinberg equilibrium using the *X*^2^ test and all the genotype distribution in controls complied with Hardy-Weinberg equilibrium. Haploview version 4.2 was used to infer LD structure and Haplotype. The association between the risk and SNPs were estimated as odds ratios (OR) and 95 % confidence intervals (95% CI) computed by logistic regression under a dominant or recessive genetic model adjusted for age and gender. Two-tailed *P* values at levels less than 0.05 were considered as significant. All the statistical analysis were carried out with the SPSS 18.0.

## SUPPLEMENTARY MATERIALS TABLES


