# Supplementary material for: Risk of eighteen genome-wide association study-identified genetic variants for colorectal cancer and colorectal adenoma in Han Chinese
Source: Oncotarget. 2016 Oct 19;7(47):77651–63. doi: 10.18632/oncotarget.12750 (PMC5363611; doi:10.18632/oncotarget.12750)
Supplement: Supplementary file 1 [file oncotarget-07-77651-s001.pdf]

Risk of eighteen genome-wide association study-identified genetic variants for colorectal cancer and colorectal adenoma in Han Chinese

SUPPLEMENTARY TABLES

Supplementary Table S1: SNPs associated with CRC or CRA risk in Chinese

location[a]	rsID	none risk / risk allele	MAF CRC	MAF CRA	Colorectal cancer				Colorectal adenoma			
					OR(95%CI)			P-value[c]	OR(95%CI)			*P*-value[c]
					Aa	aa	dominant model		Aa	aa	dominant model	
2q32.2	rs11903757[d]	T/C	0.04	0.07	1.01 (0.72-1.42)	NA[b]	NA	NA	**1.96 (1.28-3.00)**	NA	NA	0.0026
8q24.21	rs10505477	C/T	0.46	0.44	**1.50 (1.23-1.84)**	**1.56 (1.21-2.02)**	**1.52 (1.25-1.84)**	<0.0001	**1.50 (1.10-2.03)**	1.28 (0.86-1.92)	**1.44 (1.08-1.92)**	0.013
8q24.21	rs10808555	A/G	0.33	0.31	**1.26 (1.05-1.53)**	1.21 (0.89-1.64)	**1.25 (1.05-1.50)**	0.014	1.17 (0.88-1.55)	1.02 (0.64-1.64)	1.14 (0.87-1.49)	0.34
8q24.21	rs7014346	G/A	0.33	0.30	**1.29 (1.07-1.55)**	1.21 (0.89-1.64)	**1.27 (1.06-1.52)**	0.0079	1.15 (0.87-1.52)	0.93 (0.57-1.51)	1.10 (0.84-1.44)	0.47
8q24.21	rs7837328	G/A	0.44	0.39	**1.48 (1.20-1.82)**	**1.63 (1.23-2.15)**	**1.52 (1.25-1.85)**	<0.0001	1.25 (0.93-1.68)	0.98 (0.63-1.52)	1.18 (0.89-1.57)	0.24
9p24.1	rs719725	A/C	0.34	0.32	**1.27 (1.04-1.54)**	1.21 (0.89-1.64)	**1.26 (1.04-1.51)**	0.015	**1.37 (1.03-1.82)**	0.91 (0.56-1.49)	1.27 (0.97-1.67)	0.086
10q22.3	rs704017	A/G	0.29	0.25	**1.21 (1.01-1.46)**	0.95 (0.67-1.36)	1.17 (0.98-1.40)	0.079	0.92 (0.70-1.22)	0.67 (0.37-1.21)	0.88 (0.67-1.16)	0.36
10q25.2	rs11196172	A/G	0.41	0.36	**2.02 (1.66-2.46)**	**1.83 (1.31-2.56)**	**1.99 (1.65-2.41)**	<0.0001	1.23 (0.92-1.66)	1.58 (0.97-2.57)	1.29 (0.97-1.71)	0.084
15q13.3	rs4779584	C/T	0.15	0.18	**0.70 (0.57-0.86)**	0.64 (0.39-1.05)	**0.69 (0.57-0.84)**	0.0002	0.85 (0.63-1.14)	0.78 (0.38-1.61)	0.84 (0.63-1.12)	0.24
18q21.1	rs7229639	A/G	0.20	0.20	1.21 (1.00-1.47)	1.44 (0.88-2.37)	**1.23 (1.02-1.48)**	0.028	1.21 (0.91-1.61)	1.17 (0.53-2.54)	1.21 (0.91-1.60)	0.19

[a]SNP locations based on Human Genome build 36.
[b]not available
[c]Adjusted for age sex
[d]OR and 95%CI for rs11903757 TC VS TT genotype in our study

Supplementary Table S2: Comparison of our study and recent GWAS in East Asians and Chinese

rsID	MAF controls	MAF CRC	Colorectal cancer			P-value	GWAS in East Asians[a]		GWAS in Chinese 1[b]		GWAS in Chinese 2[c,d]
			OR(95%CI)								
			Aa	aa	dominant model		OR(95%CI)	P-value	OR(95%CI)	P-value	P-value
rs11903757	0.04	0.04	1.01 (0.72-1.42)	NA	NA	NA	NA	NA	0.92 (0.67-1.27)	0.614	0.588
rs10505477	0.40	0.46	**1.50 (1.23-1.84)**	**1.56 (1.21-2.02)**	**1.52 (1.25-1.84)**	<0.0001	**1.12 (1.03-1.21)**	**0.005**	**1.18 (1.05-1.32)**	**0.00665**	**0.0162**
rs10808555	0.30	0.33	**1.26 (1.05-1.53)**	1.21 (0.89-1.64)	**1.25 (1.05-1.50)**	0.014	NA	NA	NA	NA	NA
rs7014346	0.30	0.33	**1.29 (1.07-1.55)**	1.21 (0.89-1.64)	**1.27 (1.06-1.52)**	0.0079	**1.11 (1.02-1.21)**	**0.016**	**1.17 (1.03-1.33)**	**0.0136**	0.434
rs7837328	0.38	0.44	**1.48 (1.20-1.82)**	**1.63 (1.23-2.15)**	**1.52 (1.25-1.85)**	<0.0001	NA	NA	NA	NA	NA
rs719725	0.30	0.34	**1.27 (1.04-1.54)**	1.21 (0.89-1.64)	1.26 (1.04-1.51)	0.015	NA	NA	NA	NA	NA
rs704017	0.27	0.29	**1.21 (1.01-1.46)**	0.95 (0.67-1.36)	1.17 (0.98-1.40)	0.079	NA	NA	**1.14 (1.01-1.30)**	**0.0414**	**0.0493**
rs11196172	0.31	0.41	**2.02 (1.66-2.46)**	**1.83 (1.31-2.56)**	**1.99 (1.65-2.41)**	<0.0001	NA	NA	**1.32 (1.17-1.50)**	1.0000136	0.959
rs3824999	0.39	0.40	1.10 (0.91-1.34)	1.08 (0.83-1.40)	1.10 (0.91-1.32)	0.33	NA	NA	1.09 (0.97-1.23)	0.156	0.52
rs10849432	0.20	0.21	0.96 (0.79-1.16)	1.30 (0.84-2.03)	0.99 (0.83-1.19)	0.94	NA	NA	**1.18 (1.01–1.38)**	**0.0436**	0.575
rs16969681	0.41	0.41	1.01 (0.83-1.24)	1.02 (0.78-1.32)	1.02 (0.84-1.22)	0.87	NA	NA	NA	NA	NA
rs11632715	0.17	0.17	0.94 (0.77-1.14)	0.99 (0.61-1.61)	0.94 (0.78-1.14)	0.54	NA	NA	NA	NA	NA
rs4779584	0.20	0.15	**0.70 (0.57-0.86)**	0.64 (0.39-1.05)	**0.69 (0.57-0.84)**	0.0002	**1.12 (1.04-1.20)**	**0.002**	**1.23 (1.06-1.43)**	**0.00793**	0.429
rs12603526	0.26	0.27	1.11 (0.92-1.34)	0.93 (0.64-1.35)	1.09 (0.91-1.29)	0.37	NA	NA	**1.44 (1.24-1.68)**	**2.61E-06**	0.229
rs4939827	0.28	0.29	0.98 (0.82-1.18)	1.14 (0.81-1.63)	1.01 (0.84-1.20)	0.94	NA	NA	**1.16 (1.02-1.32)**	**0.0217**	0.133
rs7229639	0.17	0.20	1.21 (1.00-1.47)	1.44 (0.88-2.37)	**1.23 (1.02-1.48)**	0.028	NA	NA	**1.26 (1.09-1.45)**	0.0022	0.0826
rs4813802	0.23	0.25	1.18 (0.98-1.43)	1.15 (0.79-1.68)	1.18 (0.98-1.41)	0.074	NA	NA	NA	NA	NA
rs961253	0.08	0.08	1.03 (0.81-1.32)	0.59 (0.17-2.04)	1.01 (0.79-1.29)	0.93	NA	NA	NA	NA	NA

NA=not available
[a]Wei-Hua Jiaet et al. Nature Genetics 2013
[b]Meilin Wang et al.Nature Communications 2016
[c]Kewei Jiang et al.Oncotarget 2016
[d]OR and 95%CI of these variants are not available in this study
